# Extrahepatic Portal Vein Thrombosis, an Important Cause of Portal Hypertension in Children

**DOI:** 10.3390/jcm10122703

**Published:** 2021-06-18

**Authors:** Alina Grama, Alexandru Pîrvan, Claudia Sîrbe, Lucia Burac, Horia Ştefănescu, Otilia Fufezan, Mădălina Adriana Bordea, Tudor Lucian Pop

**Affiliations:** 1 2nd Pediatric Discipline, Department of Mother and Child, Iuliu Hațieganu University of Medicine and Pharmacy, 400112 Cluj-Napoca, Romania; gramaalina16@yahoo.com (A.G.); pirvanaaa@yahoo.com (A.P.); 2 Centre for Expertise in Pediatric Liver Rare Diseases, 2nd Pediatric Clinic, Emergency Clinical Hospital for Children, 400177 Cluj-Napoca, Romania; lucia.burac@gmail.com; 3 Hepatology Department, Regional Institute of Gastroenterology and Hepatology, 400162 Cluj-Napoca, Romania; horia.stefanescu@irgh.ro; 4 Liver Research Club, 400162 Cluj-Napoca, Romania; 5 Department of Imaging, Emergency Clinical Hospital for Children, 400078 Cluj-Napoca, Romania; otilia.fufezan@gmail.com; 6 Department of Microbiology, Iuliu Hatieganu University of Medicine and Pharmacy, 400151 Cluj-Napoca, Romania; bordea_madalina@yahoo.com

**Keywords:** portal vein thrombosis, umbilical vein catheter, thrombophilia, upper gastrointestinal bleeding, splenomegaly, children

## Abstract

One of the most important causes of portal hypertension among children is extrahepatic portal vein thrombosis (EHPVT). The most common risk factors for EHPVT are neonatal umbilical vein catheterization, transfusions, bacterial infections, dehydration, and thrombophilia. Our study aimed to describe the clinical manifestations, treatment, evolution, and risk factors of children with EHPVT. Methods: We analyzed retrospectively all children admitted and followed in our hospital with EHPVT between January 2011–December 2020. The diagnosis was made by ultrasound or contrast magnetic resonance imaging. We evaluated the onset symptoms, complications, therapeutic methods, and risk factors. Results: A total of 63 children, mean age 5.14 ± 4.90 (33 boys, 52.38%), were evaluated for EHPVT during the study period. The first symptoms were upper gastrointestinal bleeding (31 children, 49.21%) and splenomegaly (22 children, 34.92%). Thrombocytopenia was present in 44 children (69.84%). The most frequent risk factors were umbilical vein catheterization (46 children, 73.02%) and bacterial infections during the neonatal period (30 children, 47.62%). Protein C, protein S, antithrombin III levels were decreased in 44 of the 48 patients tested. In 42 of these cases, mutations for thrombophilia were tested, and 37 were positive. Upper digestive endoscopy was performed in all cases, revealing esophageal varices in 56 children (88.89%). All children with gastrointestinal bleeding received an octreotide infusion. In 26 children (41.27%), variceal ligation was performed, and in 5 children (7.94%), sclerotherapy. Porto-systemic shunt was performed in 11 children (17.46%), and Meso-Rex shunt was done in 4 children (6.35%). The evolution was favorable in 62 cases (98.41%). Only one child died secondary to severe sepsis. Conclusions: EHPVT is frequently diagnosed in the last period in our region due to the increased use of umbilical vein catheterization. Furthermore, genetic predisposition, neonatal bacterial infections, and prematurity certainly play an important role in this condition. A proactive ultrasound assessment of children with risk factors for EHPVT should be encouraged for early diagnosis and treatment.

## 1. Introduction

In children, portal hypertension (PHT) is defined as a pathological increase of the pressure in the portal system, with a pressure gradient between the portal vein and inferior vena cava greater than 5 mmHg [1]. As one of the causes of PHT in children, extrahepatic portal vein thrombosis (EHPVT) is a rare disorder, with an incidence of 1 in 100,000 live births or 1 to 36 per 1000 newborns requiring hospitalization in intensive care units. The main clinical manifestations of EHPVT are upper gastrointestinal bleeding and splenomegaly. Most of the time, these symptoms are absent during the neonatal period and appear later in childhood. For this reason, the diagnosis is established more frequently in older children [2].

The causes of EHPVT in children are not entirely known, but several factors that predispose to this pathology are described [3]. These are classified into three categories: local factors that can cause injury to the portal vein (abdominal infections, abdominal surgery, umbilical catheter), general factors (procoagulant status), and, less often, vascular malformation. The most common cause is umbilical vein catheterization (UVC), ranging from 20% in low-income countries up to 60% in developing countries [4]. Among the general factors that predispose to venous thrombosis are thrombophilia, sepsis, and dehydration. Deficiency or qualitative abnormalities of anti-coagulation factors (antithrombin III, protein C, protein S, and activated protein C resistance) often predispose to thrombotic events, including EHPVT. Thrombophilia is incriminated in 35% of cases of EHPVT in children. For this reason, children with EHPVT, and especially those that associate other risk factors (UVC), should be screened for inherited prothrombotic disorders: prothrombin 20210 mutation (PTHR), factor V Leiden (FVL), methylenetetrahydrofolate reductase (MTHFR) genes deficiency, or metabolic defects like hyper-homocysteinemia. Congenital abnormalities (portal vein stenosis, atresia, or agenesis) are rarely involved in EHPVT [5,6,7,8]. Furthermore, early EHPVT after liver transplantation with cadaveric graft was described in adults. Even less often in children, EHPVT after splenectomy for hematologic diseases was also described. An association between more than one factor is frequently observed, which further increases the risk of thrombosis. In almost 50% of cases, the etiology of EHPVT remains unknown [8].

This study aimed to describe the clinical manifestations, treatment methods, disease evolution, and analyze the main risk factors for EHPVT in children.

## 2. Materials and Methods

We performed a retrospective study including children with EHPVT followed-up in the 2nd Paediatric Clinic of the Emergency Clinical Hospital for Children Cluj-Napoca, the leading pediatric gastroenterology and hepatology center in Transylvania, Romania, during a period of 10 years (January 2011–December 2020).

The inclusion criteria were represented by children under 18 years of age with a definite diagnosis of EHPVT. We excluded from the study children with the other causes of PHT (cirrhosis, congenital liver fibrosis, Budd-Chiari syndrome, or malignancies), patients with ambiguous diagnosis, incomplete data, or those lost from the follow-up.

The diagnosis of EHPVT was based on the clinical picture (upper digestive hemorrhage and splenomegaly), clinical history data, and ultrasound changes highly suggestive for PHT diagnosis (splenomegaly, enlargement of the portal vein and its tributaries, or the visualization of the portal obstruction or neovascularization, cavernoma). Portal thrombosis was described as a heterogeneous material in the vessel lumen and less often isoechoic or hypoechoic. The interpretation of the images and the measurements were performed by the same team of radiologists from our hospital.

We analyzed the demographic characteristics (age and gender), clinical manifestations (onset symptoms, clinical exam, and complications) and laboratory and imaging characteristics, the role of the therapeutic methods, and the outcome. We also studied data related to patient history: gestational age, birth weight, infection presence in the neonatal period, umbilical catheterization, dehydration, and the history of thromboembolism. Complete blood count, coagulation parameters, upper endoscopy, and abdominal Doppler ultrasound were performed in all patients. In 48 children, we measured the level and the activity of protein C (PC), protein S (PS), antithrombin III (ATIII), or homocysteine. Proteins activity was measured by coagulometric assay and was expressed as a percentage of a reference plasma level. In 42 cases, we performed the genetic test for thrombophilia, respectively: mutations for Factor V G1691A (Leiden), Factor V H1299R (R2), Factor II G20210A, C677T methylenetetrahydrofolate reductase (MTHFR-C677T), Factor XIII V34L, Endothelial Protein C Receptor (EPCR), and PAI-1 4G/5G. Genetic tests were performed using polymerase chain reaction technique (PCR)—restriction fragment length polymorphism and hybridization methods.

Regardless of the onset of symptoms, upper digestive endoscopy was performed in all children, either at the first admission or during the follow-up. Varices were classified according to Baveno guidelines: grade I (small varices, <5 mm), grade II (medium caliber varices), and grade III (large caliber or varices, >5 mm) [9].

The patients were monitored every 3 to 6 months by clinical examination, basic laboratory tests, color Doppler ultrasound examination, or upper digestive endoscopy (when needed). In addition, when required, endoscopic treatment (variceal band ligation or sclerotherapy) was carried out in the Endoscopy Unit of the Regional Institute of Gastroenterology and Hepatology, Cluj-Napoca, Romania.

This study was carried out according to the Declaration of Helsinki principles after obtaining informed consent from the parents.

All data were analyzed statistically using the Statistica software, Version 13 (TIBCO Software Inc. Palo Alto, CA, USA). We used descriptive statistics for continuous variables (means and standard deviations, SD) and *t*-Student test for statistical significance testing (age, white blood cells, hemoglobin level, and platelets). We used the Pearson’s chi-square value, calculating the Pearson-r coefficient for the correlations between qualitative variables (sex, risk factors, presence and severity of varices, and treatment between patients with upper gastrointestinal bleeding, splenomegaly, or ultrasound exam as clinical presentation of EHPVT). The results were considered statistically significant at values of *p* < 0.05.

## 3. Results

We included in our study 63 patients with age at the diagnostic from 6 months to 17 years and 10 months (mean age of 5.14 ± 4.90 years). There were 33 boys (52.38%) and 30 girls (47.62%).

Clinical features at the first presentation were hematemesis and melena in 31 patients (49.21%). Patients’ age at the time of the first upper gastrointestinal bleeding was between 2 years and 16 years and 2 months (mean age of 9.47 years). There were 40 children (61.90%) with bleeding during disease evolution: 20 children (33.33%) with 1 episode, 5 children (7.93%) with 2 episodes, 3 children (4.76%) with 3 episodes, and in 11 children (17.46%), more episodes. In 32 patients (50.79%), the onset of disease was without bleeding: splenomegaly was the first sign in 22 children (34.92%), and in 10 children (15.87%), the diagnostic was established by ultrasound examination.

There were 7 children (11.11%) without varices at the first endoscopic evaluation, and 56 (88.89%) with varices of different grades: 9 children (14.29%) with small varices, 19 children (30.16%) with medium varices, and 28 children (44.44%) with large varices. In 14 children (22.22%), there were also gastric varices. On physical examination, 54 (85.71%) presented splenomegaly at the first evaluation, subsequently confirmed by ultrasound. Thrombocytopenia (<150,000/mm^3^) was reported in 44 children (69.84%). The mean platelet count was 142,587 ± 100,158/mm^3^.

Regarding the risk factors for EHPVT, 61 children presented at least one known risk factor (96.83%). UVC was used during the neonatal period in 46 children (73.02%). Bacterial infections during the neonatal period were present in 30 children (47.62%), and dehydration in 12 children (19.08%). Regarding bacterial infections, seven children were with acute respiratory distress syndrome (ARDS) on acute lung infection, six were with gastrointestinal sepsis, four with septic complications after surgery for digestive malformations, and three for cardiac malformation, one was with meningitis with *Streptococcus B*, and nine had incomplete data on this issue. Administration of transfusions on the umbilical catheter also increases the risk of thrombosis. In our cohort, this association was observed in 10 children (15.87%). Of 48 children tested for coagulation abnormalities, decreased PS, PC, or ATIII serum level was observed in 44 patients (91.67%). In 42 children, we performed genetic tests for thrombophilia, and in 37 children (91.30%) these were positive. Abdominal surgery for congenital malformation was performed in six (9.52%) children in our study. Other frequent predisposing factors identified in our cohort were prematurity (28 children; 44.44%) and low birth weight (24 children; 38.10%).

Comparing the presence of these risk factors with the onset of EHPVT, in our cohort, only the use of UVC was more frequently associated with upper gastrointestinal bleeding than with the other forms of presentation (*p* = 0.00011, Table 1). Considering all these risk factors, 13 children (20.63%) had only 1 risk factor, 11 children (17.46%) had 2 risk factors, 12 children (19.05%) had 3 risk factors, and 25 children (39.68%) were associated with 4 or more risk factors. In the group of children with only one risk factor identified, seven were with genetic mutations for thrombophilia, four with UVC, and one with previous abdominal surgery. There was no statistically significant correlation between the number of risk factors identified in one patient and the clinical presentation of EHPVT.

Octreotide infusion was used for the management of acute variceal bleeding along with endoscopic therapy. Of all 88 hospitalizations for upper digestive bleeding, in 58 episodes (65.90%), octreotide therapy was enough to stop the bleeding. In the other episodes, emergency endoscopic band ligation (26 children, 41.27%) or sclerotherapy with Glubran 2^®^ (GEM; Viareggio, Italy), N-butyl-2 cyanoacrylate plus methacryloxysulfolane (5 children, 7.94%) was necessary. Sclerotherapy was used in patients who also bled from their gastric varices. The recurrence rate of bleeding in children after endoscopic treatment was 10% (three children). In 35 episodes of gastrointestinal bleeding, blood transfusions were administered to correct anemia.

Regarding primary and secondary prophylaxis, 60 children (95.24%) received β-adrenergic antagonists. Propranolol was used as primary prophylaxis at a dose adjusted to reduce heart rate by 25%. In 11 children (17.46%), endoscopic variceal band ligation or sclerotherapy was performed. Due to recurrent gastrointestinal bleeding or severe thrombocytopenia, surgical treatment was performed in 15 children (23.81%). In six patients (9.52%), a distal splenorenal shunt was performed; in two patients (3.17%) mesorenal shunt; and in three patients (4.76%) mesocaval shunt. In four children (6.34%), meso-Rex bypass (with jugular vein) was successfully made. Of all the patients who received surgical treatment, five cases presented complications (thrombosis), two of them with proven thrombophilia. Of those five, only one patient remained without shunt patency and presented gastrointestinal bleeding one year after shunting. No patient received liver transplantation.

In our cohort, 18 children (29.51%) were recorded as underweight (−2 SD for weight for age) during the follow-up.

## 4. Discussion

EHPVT is a common cause of PHT in children and adolescents in our region. Although a rare condition, the number of cases continues to grow in our area due to the increasing use of the UVC on neonatal intensive care units. In most cases, there is an association of risk factors responsible for thrombosis. Local prothrombotic factors, as UVC, can be associated with prematurity. UVC is needed in these patients when the venous approach is challenging. Other predisposing factors identified were low birth weight and dehydration. Most authors describe the role of UVC in the pathogenesis of EHPVT. In a study from Toronto, Canada, among the 133 neonates who underwent UVC, 72.93% developed EHPVT [5,6]. Furthermore, UVC was the main etiological factor of EHPVT (65%) in a group of 187 Italian children, 61% of them being premature [4]. In Brazil, the history of UVC was present in 40.6% of children with EHPVT [10]. Administration of hypertonic substances or highly irritating drugs on these catheters can cause endothelial damage, inflammation, fibrosis, and thrombus formation, with blood flow obstruction and neovessels formation (cavernoma). In addition, its use for extended periods as well as the association of other general risk factors (procoagulant status) or local factors (dehydration, abdominal infections, or abdominal surgery) will further increase the risk of EHPVT especially in premature neonates [11,12].

Sepsis was the second most important risk factor for EHPVT in our cohort, but also local infections may be involved. Although more common in adults, pylephlebitis is also described in children as a predisposing condition for EHPVT. Pylephlebitis may be due to omphalitis and intra-abdominal infections (ulcero-necrotic enterocolitis in premature neonates or diverticulitis, appendicitis, cholecystitis, and pancreatitis in the older child). Staphylococcus and Gram-negative bacilli are most commonly described as the pathogens involved in pylephlebitis. Furthermore, fungal infections may cause this septic thrombophlebitis. The involvement of abdominal infection in the development of EHPVT was first reported in 1927 by Wallgreen and was later developed by other authors [2]. In a study of Kanellopoulou, of 100 patients (children and adults) with acute or chronic pylephlebitis, EHPVT occurred in 81 cases [13]. Along with this study, the author suggested the benefits of anticoagulant therapy in these cases [14]. Another team of researchers led by Nayman identified sepsis as a risk factor in 70% of patients who developed EHPVT [15].

Coagulation disorders often predispose to EHPVT, mainly if they are associated with local factors. Abnormal values of the anti-coagulation proteins were observed in a significant number of our cases. It is challenging to specify to what degree the reduction of these proteins level is due to a genetic deficit (thrombophilia). We already know that protein C or S deficiency may be associated with EHPVT in children and adolescents without being proved to be the determining factor. The frequency of protein C, protein S, or antithrombin III deficiency is estimated at 40–50% of EHPVT cases. In our study, it was even higher. The decreased level of these proteins can be secondary to the thrombosis itself, as reported by some authors, or can result from their consumption in portosystemic shunts. The neovascular formations (cavernoma) will cause a hepatopetal flow that is not enough to reduce the pressure, and spontaneous natural portosystemic shunts may be formed. These shunts function as “release valves” to reduce the pressure in the portal space. But this compensatory mechanism is insufficient and does not allow adequate reduction of portal pressure, thus causing the consumption of these anticoagulant proteins. Another cause of protein C or S deficiency is the hepatic injury caused by reduced flow through the portal vein [16]. In children with anti-coagulation protein deficiency, we performed genetic tests, and the number of positive results was significant (over 80% of those tested). The incidence of inherited thrombophilia is described as between 30% and 70% of EHPVT patients. At Bambino Gesu Children’s Hospital in Roma, Italy, from 31 cases with EHPVT, 32.3% had inherited thrombophilic abnormalities [17]. It is difficult to evaluate the genetic predisposition for thrombophilia as a routine test in children at their presentation in many countries. If possible, these analyses would help to explain the factors that may lead to EHPVT but are most commonly associated with other existent factors. In our cohort, almost 80% of children were associated with two or more risk factors. Even in those with only one risk factor identified, just half of them had genetic mutations for thrombophilia as a risk factor.

Other risk factors for EHPVT in our study were vascular anomalies and abdominal surgery for congenital malformations. The prevalence of abdominal surgery as a cause of EHPVT was similar to other studies (8–20%). In our study, five children had abdominal surgery and then developed EHPVT: a boy with hepatoportal aneurysm, three children with lower gastrointestinal malformations, and one boy with coarctation of the abdominal aorta who developed numerous clamps requiring enterectomy. All these interventions, associated or not with other risk factors, have favored over time the thrombosis.

The most common clinical manifestations were upper digestive hemorrhage and splenomegaly in our patients. EHPVT is a significant cause of upper gastrointestinal bleeding in children and adolescents. In our children, the most frequent presentations were hematemesis and/or melena, frequently triggered by nonsteroidal anti-inflammatory drugs (NSAIDs) use. In fact, in most studies, upper gastrointestinal bleeding was the main clinical presentation in children with EHPVT (over 50%), followed by splenomegaly (around 35%). According to other authors, about 79% of children with EHPVT will develop at least one episode of upper gastrointestinal bleeding during their lifetime [18]. Gastrointestinal bleeding in children with EHPVT is better tolerated than those with cirrhosis due to intact liver function; the mortality rate due to bleeding in EHPVT is lower than in cirrhosis (2-5%) [8]. In older children and teenagers, the risk of variceal bleeding decreases due to the development of spontaneous portosystemic collaterals. The second frequent clinical presentation of EHPVT in our children was splenomegaly, with its consequences (thrombocytopenia, leukopenia, or anemia secondary to hypersplenism). Ascites, jaundice, or hepatic encephalopathy are described in the literature as possible onset manifestations in EHPVT patients, but these manifestations were not presented in our cohort.

A complication encountered in our children was growth retardation. The causes of growth retardation in children with EHPVT are not entirely known today. Several factors are described: intestinal malabsorption secondary to venous stasis, anorexia secondary to splenomegaly, or fear of bleeding. Moreover, growth retardation in EHPVT is secondary to resistance to growth hormone action and reduction of hepatotrophic hormone synthesis due to decreased hepatic vascularization [19,20]. In our children, the weight deficit was mild or moderate, which allowed an effective intervention for nutritional recovery.

For the EHPVT diagnosis, an abdominal Doppler ultrasound exam is the method of choice. We used mainly this method and less the other invasive investigations such as angiography. Detailed ultrasound and endoscopic monitoring of these children is critical, given the presence of esophageal varices in patients who never have bled. The prevalence of ectopic varices in these cases is higher than in cirrhotic children [21]. For patients with EHPVT, the degree of esophagogastric varices is directly related to the incidence of upper gastrointestinal bleeding. Furthermore, the risk of digestive bleeding increases with the number of previous bleeding episodes. Fortunately, portal hypertensive gastropathy is rare, but it worsens the prognosis significantly [22].

To date, there is no universally accepted follow-up and treatment protocol for children with EHPVT. According to some authors, early administration of anticoagulants (low-molecular-weight heparin) in the neonatal period in children who develop EHPVT is effective, while others oppose these measures. Although anticoagulant therapy has proven effective in adults, causing re-permeabilization of the portal vein in up to 70% of cases, in children, the risk of complications is higher due to young age and comorbidities. However, there is no guarantee that this therapy will lead to remission of the thrombotic process. Furthermore, anticoagulant therapy should be initiated promptly, preferably in the first week after the onset of the acute thrombotic process, which is difficult because the thrombus is rarely visualized so quickly in neonates. Furthermore, with the cavernoma formation, the recanalization of the portal vein becomes unlikely despite therapy, and there is a high rate of variceal bleeding. Therefore, the use of anticoagulants in children remains controversial [2,21]. In a study including 244 neonates, all 15 who developed EHPVT received low-molecular-weight heparin after thrombus detection by Doppler ultrasound. In eight children, this was resolved, and in the other seven cases, thrombosis persisted [22]. The experience with this therapy is relatively poor in children. In a study including 113 neonates who received anticoagulant treatment due to the presence of risk factors (UVC, sepsis, asphyxia, dehydration, and thrombophilia), 51 of them developed EHPVT anyway [23]. However, no patient was receiving anticoagulant treatment in our cohort.

In EHPVT, treatment goals include emergency measures to stop variceal bleeding, prevent the first episode of bleeding (primary prophylaxis) and recurrent variceal bleeding (secondary prophylaxis). Treatment includes medical or endoscopic measures. The urgency in EHPVT is the treatment of variceal bleeding, which occurs in approximately 70% of children [8]. We used vasoactive agents such as octreotide and variceal band ligation or sclerotherapy.

Medical treatment for EHPVT includes beta-blockers for primary prophylaxis. In adults, the utility of beta-blocker medication (propranolol or nadolol) for the primary prophylaxis of variceal bleeding has been established in long-term reports. However, the widespread use of this medication as primary prophylaxis in children is controversial [24]. In a retrospective study conducted in Boston, the United States, only 33% of all children who received treatment with propranolol for EHPVT presented upper gastrointestinal bleeding in the next three years [25]. A study from Turkey also sustains the usefulness of this therapy. Out of 45 children with EHPVT treated with propranolol, 8 children (15.6%) presented upper gastrointestinal bleeding during primary prophylaxis [25]. Most studies report a bleeding rate among those who received treatment with propranolol between 2% and 11% per year of follow-up [26]. According to other authors, the degree of varices decreased with beta-blocker therapy, but the results do not reflect the usefulness of their use in children [27]. Hence the effectiveness of non-selective beta-blockers for the prevention of esophageal varices in children with PHT remains unclear. The decision to administer or not depends on the experience of each center. In our center, we used propranolol treatment in a significant number of cases. Still, it is difficult to assess how this medication has influenced the evolution of the patients or not.

Sclerotherapy and variceal band ligation are essential treatment methods both in severe life-threatening bleeding and in preventing possible bleeding in those with high-grade varices. Children with large varices should be considered for primary prophylaxis with endoscopic therapy. Almost half of our children had variceal ligations and sclerotherapy, the last being used mainly in cases with bleeding from gastric varices. Emergency endoscopic variceal ligation is a highly effective method for stopping acute bleeding (>90% of cases), being preferred due to the lower risk of complications or rebleeding. The main impediment of this method is the recurrence of esophageal varices when sclerotherapy is recommended. In a large study including 101 children with EHPVT and acute variceal bleeding, endoscopic ligation followed by sclerotherapy leads to varices eradication in over 98% of cases [28]. These methods also have complications, including rebleeding, ulceration, stricture, perforation, or systemic side effects secondary to sedation methods [29]. Generally, the recurrence rate of bleeding in children after endoscopic treatment is low (4–14%), confirmed by our study [8]. Fortunately, as far as we are concerned, these complications were limited to a relatively small number of cases: two episodes of cardio-respiratory arrest secondary to sedation in one of our patients.

Children with recurrent bleedings despite these measures will be suitable for surgical treatment (shunts or bypass). The portosystemic shunts may reduce the pressure on the entire splanchnic territory (portacaval, mesocaval, or proximal splenorenal shunts) or decompress only the gastroesophageal veins while the flow from the superior mesentery remains constant (distal splenorenal shunt). The role of portosystemic shunts is well known in controlling variceal bleeding and symptoms related to hypersplenism. The main indications for surgical treatment include severe bleeding that endoscopic measures cannot control, persistent high-grade varices, important splenomegaly with significant thrombocytopenia, or impaired growth [30,31,32,33]. The choice of shunt type depends on several factors, including the child’s general condition, the association of other comorbidities, the vascular anatomy, or the resources and experience of the surgical team [1]. In our study, the most commonly performed was splenorenal shunt, followed by mesocaval and mesorenal shunt. The evolution was favorable in most of the cases. Only a few children developed complications as total or partial stenosis of the shunt. Another surgical procedure with excellent results is the mesenteric-left portal bypass (Meso-Rex bypass). This procedure aims to restore the portal vein’s flow by creating an anastomosis between the superior mesenteric vein and the left portal vein. This procedure was used for the first time for portal vein thrombosis after liver transplantation. This method is used in children with EHPVT with a weight/portal vein diameter ratio greater than 10. The meso-Rex shunt reduces the portal vein pressure, the degree of esophageal and gastric varices, or the splenomegaly and significantly improved the prognosis in children with EHPVT. Doppler ultrasonography has shown an excellent intrahepatic portal flow after Rex-bypass shunt [34,35,36].

The transjugular intrahepatic portosystemic shunt (TIPSS) is a non-surgical method by which a metallic stent is inserted between the hepatic vein and intrahepatic portal vein. The experience of TIPSS in children is limited to several case reports or small series with complications that can hamper the feasibility of other surgical procedures. TIPSS may be used when surgical shunting was contraindicated or as a bridge to liver transplantation. The decision to use this procedure in children should be based on a thoughtful analysis of benefits and risks for each patient [37,38].

## 5. Conclusions

Currently, EHPVT is a common cause of severe upper gastrointestinal bleeding or splenomegaly in children from the Nord-Western of Romania. Our study relates the importance of risk factors such as UVC, procoagulant status, or bacterial infections in the development of EHPVT in children. A deeper understanding of the risk factors in developing thrombosis could provide the basis for new therapeutic strategies. There is a need for a management protocol and preventive approach for children with EHPVT to improve their outcomes.

## Figures and Tables

**Table 1:** Comparison between children with EHPVT depending on the clinical presentation.

Clinical Features at the First Presentation	Total	Upper Gastrointestinal Bleeding	Splenomegaly	Ultrasound Examination	*p*-Value
Number (%)	63 (100%)	31 (49.21%)	22 (34.92%)	10 (15.87%)	-
Age, years	5.26 ± 4.94	4.40 ± 4.15	4 ± 5.65	6.92 ± 5.38	-
Males, *n* (%)	33 (50.79%)	19 (54.84%)	9 (52.38%)	5 (36.36%)	0.3379
Risk factors:					
UVC	46 (72.02%)	30	12	4	0.00011
Thrombophilia	37/42 * (91.67%)	16	14	7	0.51769
Bacterial infections	30 (47.62%)	17	9	4	0.52792
Preterm birth	28 (44.44%)	16	6	6	0.32009
Low birthweight	24 (38.10%)	15	4	5	0.05806
Dehydration	12 (19.08%)	7	3	2	0.71369
Transfusions on UVC	10 (15.87%)	5	4	1	0.84045
Abdominal surgery	6 (9.52%)	3	1	2	0.38533
No risk factors identified	2 (3.17%)	0	2	0	0.14591
White blood cells, /mm^3^	5949 ± 2666	6318 ± 2810	5977 ± 2696	4740 ± 1856	NS
Hemoglobin, g/dL	11.23 ± 2.24	10.75 ± 2.70	11.66 ± 1.55	11.74 ± 1.78	NS
Platelets, /mm^3^	142,587 ± 100,158	159,581 ± 121,178	128,255 ± 77,093	119,900 ± 65,349	NS
Varices:					0.00019
- no varices	7	0	5	2	
- grade I	9	0	7	2	
- grade II	19	9	7	3	
- grade III	28	22	3	3	
Treatment:					
- Octreotide infusion	58/88 **	58	0	0	
- Endoscopic band ligation	26	20	3	3	0.00976
- Sclerotherapy	5	5	0	0	0.06062
- Surgical treatment	15	9	6	0	0.29249

* Thrombophilia tests were only performed in 42 children. ** There were 88 episodes of upper digestive bleeding. NS, non-significant; UVC, umbilical vein catheter. Data are given as mean ± SD or number of cases (percentage).

## Data Availability

Data are available on request from the corresponding author.
